# Laboratory specimen rejection rate and associated factors among referred specimens at Debre Markos Referral Hospital, Ethiopia: prospective cross-sectional study

**DOI:** 10.11604/pamj.2024.47.112.33795

**Published:** 2024-03-07

**Authors:** Bewket Mesganaw, Fatuma Hassen, Habtamu Molla, Ketema Misganaw

**Affiliations:** 1 Department of Medical Laboratory Sciences, College of Health Sciences, Addis Ababa University, Addis Ababa, Ethiopia,; 2 Ethiopian Public Health Institute, Addis Ababa, Ethiopia

**Keywords:** Specimen rejection rate, clinical laboratory errors, laboratory quality management system

## Abstract

**Introduction:**

laboratory errors mostly emerge from the pre-analytical phase, mainly those related to collection, handling, transportation, and storage of diagnostic specimens. Specimen rejection due to improper sample collection, may lead to poor patient outcomes, such as incorrect diagnosis, inappropriate treatment, and death. This study aimed to assess the specimen rejection rate and associated factors among referred specimens at Debre Markos Referral Hospital.

**Methods:**

a prospective cross-sectional study design was applied from January 2020 to April 2020 to investigate specimen rejection rate and associated factors among referred specimens. The study population was all laboratory specimens referred for viral load, CD4 count, gene expert, and early infant diagnosis to the Debre Markos Referral Hospital laboratory. The statistical analysis was done with Statistical Package for Social Sciences version 20.0 software.

**Results:**

of the total of 2750 specimens submitted to the laboratory from January 2020 to April 2020, 37 (1.34%) specimens were rejected due to different reasons like insufficient volume, hemolysis, and an inappropriate specimen container. Specimen collector training status and experience had a significant association with the specimen rejection rate.

**Conclusion:**

the results of our study show that the specimen rejection rate among referred specimens was high, indicating that more interventions are required to decrease the specimen rejection rate.

## Introduction

Clinical laboratories are the fundamental part of all health care systems. Reliable and timely results from laboratory investigations are important elements in decision-making in all aspects of health services and disease prevention programmes. To ensure good treatment for patients, quality laboratory services are mandatory by establishing and maintaining a quality management system for all activities of laboratory services, which includes an arrangement for laboratory requests, patient identification, collection of samples, transportation, storage, processing, and examination of clinical samples, interpretation of results, and reporting [1,2].

Laboratory errors, which are any defects in the laboratory testing process (pre-analytical, analytical, and post-analytical), may negatively affect the clinical decision-making process and hence result in poor patient care. Several studies have shown that most laboratory errors emerge from the manual activities of the pre-analytical phase, mainly those related to collection, handling, transportation, preparation, and storage of diagnostic specimens. Therefore, the quality of laboratory results is good if the quality of specimen collection and transportation is assured [3-5]. Proper sample collection is an important element of good laboratory practice, whereas improper sample collection may lead to poor patient outcomes, such as incorrect diagnosis, inappropriate treatment, and death. Due to inadequate materials, financial and trained personnel in most health facilities in resource-limited countries like Ethiopia, most samples are collected from peripheral laboratories. Then, it is transported to the reference laboratory for subsequent laboratory testing. In such cases, the reference laboratory should have a documented procedure for monitoring the transportation of samples to ensure they are transported within a time frame, within the temperature interval and with the required preservatives to ensure the integrity of samples [1,6]. According to the International Organization for Standardization (ISO), reference clinical laboratories should develop criteria for acceptance or rejection of specimens. Problems with patient identification, sample instability due to inappropriate sample container, insufficient sample volume, clotted samples, and hemolyzed samples are some of the examples of rejection criteria [1,6].

Improper collection, handling, and transportation of specimens may also lead to specimen rejection. Those patients whose specimens are rejected are mostly subjected to repeated specimen collection, which results in inconvenience and discomfort from repeated phlebotomy. Rejection of specimens and the need for recollection of specimens may lead to a delay in laboratory analysis. The prolonged turnaround time, especially for referred specimens, has a significant impact on the patient´s health outcome [7]. To increase access of the community with adequate and quality specialized laboratory services, feasible and applicable linkage mechanism among laboratories has been established in Ethiopia since 2008. In the implementation of a referral system, specimens are collected from any health facility and transported via a suitable courier system to referral laboratories where the testing service is available [8].

Debre Markos Referral Hospital laboratory is one of the referral laboratories to which the surrounding health facilities refer diagnostic specimens for laboratory testing, especially for viral load, gene expert for tuberculosis (TB), early infant diagnosis (EID) for HIV test and CD4 test. However, there was no study conducted in the area that shows the rate of specimen rejection and the main factors that cause the specimen to be rejected. Knowing the specimen rejection rate and the factors that cause the laboratory specimens to be rejected is important for the stakeholders to take interventions to ensure quality laboratory services. Therefore, the study aimed to determine the specimen rejection rate and the factors of rejection among referred specimens at the Debre Markos Referral Hospital laboratory.

## Methods

**Study area, design and participants:** the study was conducted at the Debre Markos Referral Hospital laboratory. The Debre Markos Referral Hospital is located in Debre Markos town, which is 300 km from Addis Ababa and 256 km from Bahir Dar. Viral load, early diagnosis (EID) for HIV/AIDS, TB gene expert, and CD4 count are not only limited to patients coming to the hospital, but also referred samples from different health facilities are tested. The Debre Markos Referral Hospital laboratory is linked to 56 health facilities to form a referral network. These are 9 district hospitals, 46 health centers, and 1 private clinic. A prospective cross-sectional descriptive study design was conducted from January 2020 to April 2020. The sampling technique was a convenient sampling technique. The study population was all laboratory specimens referred for viral load, CD4 count, gene expert, and EID to the Debre Markos Referral Hospital laboratory within the period from January 2020 to April 2020.

**Data collection procedure and analysis:** a data collection checklist was created based on ISO 15189, 2012 guidelines. We have developed two types of data collection checklists. One was developed to collect the information about the socio demographic characteristics of the specimen collector and they filled it at the referring sites. The filled data collection checklist was transported by postal services and submitted to the reference laboratory with the specimen. The other type of checklist was developed as to collect sample rejection information from the sample receipt and rejection logbook when samples were submitted to the reference laboratory. This data collection tool includes the parameters like sample type, laboratory test requested, and reasons for rejection. After training and brief instruction of the data collectors, the data were collected using the prepared data collection tool at the reference laboratory. Then the data were transferred to the Statistical Package for Social Sciences (SPSS) 20.0 software. The number of rejected samples relative to the total number of samples submitted was calculated to determine the overall rate of rejected samples. Other descriptive statistics were also compiled to show the reasons and problems associated with sample rejection among the referred samples.

**Ethical consideration:** to conduct this study, Ethical clearance was obtained from the Department Research and Ethics Committee (DREC) of Addis Ababa University. The letter of permission was also obtained from the East Gojjam zonal health department and Debre Markos Referral Hospital where the study was conducted. Written informed consent was also made with the responsible body of Debre Markos Referral Hospital laboratory and referring health facilities to assure the confidentiality of information during data collection.

## Results

**Specimen rejection rate:** in our cross-sectional study, a total of 2750 specimens were submitted from 56 health facilities (9 hospitals, 46 health centers, and 1 private clinic) to the Debre Markos Referral Hospital laboratory within the period January 2020 to April 2020, of which a total of 37 (1.34%) specimens were rejected. Among the total specimens submitted to the reference laboratory, the majority of specimens 2581 (93.8%) were plasma specimens referred for viral load testing. The highest specimen rejection among the total referred specimens also were plasma specimens referred for viral load testing (1.24%) (Table 1).

**Table 1:** specimen rejection rate among referred specimens from January 2020 to April 2020 at Debre Markos Referral Hospital, Debre Markos, Ethiopia

Status of specimen	Requested laboratory test type				Total
	Viral load	Early infant diagnosis (EID)	Gene expert	CD4 count	
Total specimens referred	2581	53	101	15	2750
Rejected specimens	34	1	1	1	37
Specimen rejection rate (%)	1.32	1.89	0.99	6.67	1.34

**Reason for rejection:** insufficient volume (37.8%), hemolysis (21.6%), and an inappropriate specimen container (13.5%) were the main reasons for specimen rejection among the referred specimens. Further evaluation of data shows that the majorities (41.2%) of the rejected specimens referred for viral load laboratory test were because of insufficient volume. The other main reasons for viral load specimen rejection were hemolysis (23.5), inappropriate specimen container (14.7%) and uncentrifuged specimen (11.8%). On the other hand, 1 specimen due to not maintaining cold chain, 1 specimen due to specimen without request paper and 1 specimen due to clotted were rejected referred for EID, gene expert and CD4 count respectively (Table 2).

**Table 2:** specimen rejection reasons in each referred test types from January 2020 to April 2020 at Debre Markos Referral Hospital, Debre Markos, Ethiopia

Reason of rejection	Requested tests				Total
	Viral load	Early infant diagnosis (EID)	Gene expert	CD4 count	
Insufficient volume	14(41.2%)	0(0%)	0(0%)	0(0%)	14(37.8%)
Hemolysis	8(23.5%)	0(0%)	0(0%)	0(0%)	8(21.6%)
Inappropriate specimen container	5(14.7%)	0(0%)	0(0%)	0(0%)	5(13.5%)
Uncentrifuged	4(11.8%)	0(0%)	0(0%)	0(0%)	4(10.8%)
Repeating label	2(5.9%)	0(0%)	0(0%)	0(050	2(5.4%)
Unlabeled specimen	1(2.9%)	0(0%)	0(0%)	0(0%0	1(2.7%)
Specimen without request paper	0(0%)	0(0%)	1(100%)	0(0%)	1(2.7%)
Clotted specimen	0(0%)	0(0%)	0(0%)	1(100%)	1(2.7%)
Not maintained cold chain	0(0%)	1(100%)	0(0%)	0(0%)	1(2.7%)
Total	34(100%)	1(100%)	1(100%)	1(100%)	37(100%)

**Factors associated with specimen rejection:** for the evaluation of specimen rejection with associated factors, we considered different factors like transport conditions, specimen collector profession, educational level, training status, and experience. Of those factors, only training status and experience of specimen collector had a significant association with specimen rejection. Evaluation of data showed that 1554 specimens were collected by trained personnel of which only 6 (0.38%) specimens were rejected, and 1196 specimens were collected by untrained personnel of which 31 (2.59%) specimens were rejected. In addition, the specimen rejection rate was higher in specimens collected by less experienced health personnel (below 1 year) than in specimens collected by more experienced health personnel (over 1 year) (Table 3).

**Table 3:** associated factors of specimen rejection from January 2020 to April 2020 at Debre Markos Referral Hospital, Debre Markos, Ethiopia

Factors	Specimen status		COR (95%CI	AOR (95% CI)	P- value
	Number of rejected specimens	Rate (%)			
**Training status**					
Not trained	31(1196)	2.59	4.023(2.446-10.247)	3.528(1.330-9.358)	0.011
Trained	6(1554)	0.34	1		
**Educational level**					
Diploma	23(1452)	1.58	2.643(1.872-4.231)	1.328(0.670-2.635)	0.417
Degree	14(1298)	1.08	1		
**Experience**					
< 1 year	15(214)	7.0	10.343(5.466-35.443)	8.917(1.055-35.394)	0.045
1-3 years	16(312)	1.2	4.221(2.121-12.321)	2.159(0.271-17.177)	0.467
3-5 years	5(904)	0.5	6.922(2.362-12.112)	1.313(0.150-11.491	0.806
>5 years	1(320)	0.3	1		

COR- crude odd ratio; AOR: adjusted odd ratio

In this study, the outcome of the final multiple logistic regression model indicates that factors like training status and experience had a significant association with specimen rejection (P = 0.01 and P = 0.04 respectively). However, the educational level of the specimen collector had no significant association with specimen rejection (P = 0.417), which has a P-value greater than 0.05. The analysis indicates that specimens collected by untrained health personnel were rejected 3.528 times more frequently than those specimens collected by trained health personnel (AOR: 3.528, 95%CI: 1.330-9.358, P = 0.01). In the same manner, specimens collected by health personnel with work experience of less than 1 year were rejected 8.917 times more frequently than those specimens collected by health personnel with work experience of more than 5 years (AOR: 8.917, 95% CI: 1.055-35.394, P=0.04). Specimens collected by health personnel with work experience of 1-3 years were rejected 2.159 times more frequently than those specimens collected by health personnel with work experience of 5+ years, and specimens collected by health personnel with work experience of 3-5 years were rejected 1.313 times more frequently than those specimens collected by health personnel with experience of 5+ years. This indicates that as experience of specimen collectors increases, the rate of specimen rejection is decreased (Table 3).

## Discussion

We conducted this study to assess the total specimen rejection rate and the factors of rejection among referred specimens for HIV viral load, early infant diagnosis (EID), gene expert for TB, and CD4 count. Hence, our finding showed that the overall specimen rejection rate is 1.34% and needs more intervention because the standard specimen rejection rate is 0.3% [9]. The finding of our study is almost similar to the specimen rejection rate determined as 1.46%, 1.44% and 1.4% in a retrospective study at Moresby General Hospital, a tertiary laboratory in Cape Town and a cross-sectional study at Addis Ababa St. Paul´s hospital, millennium medical college, respectively [10-12].

But our finding (1.34%) is lower than a retrospective study conducted in the laboratory of a tertiary care medical center in the developing world and a cross-sectional retrospective study conducted at Mutare Provincial molecular diagnosis laboratory, which was 7.2% and 10.7%, respectively [13,14]. This difference may be due to the difference in study design, the number of data collection sites, and the variety of specimen types included in the study. For example, the latter study conducted at Mutare Provincial molecular diagnosis was done only on specimens requested for early infant diagnosis (EID).

In our study, the specimen rejection rate of 1.34 percent is higher than the specimen rejection rate (0.11%) determined by a cross-sectional study at Beijing Hospital, China. This difference may be due to the previous study being conducted on only haematology specimens, whereas our study included more than one specific type of specimen. In addition, a similar study on referred specimens conducted at Amhara Public Health Institute determined the specimen rejection rate as 0.5%, which is lower than the specimen rejection rate determined by our study. This difference may come from the difference in the sample size of the two studies [5,15].

Our study showed the majority of specimens (almost 38%) were rejected due to insufficient volume. In the same manner, a cross-sectional study done in the National Microbiology Reference Laboratory of Zimbabwe showed that the main reason for specimen rejection was insufficient volume, which accounts for 72% of the total rejected specimens [14,16]. The other main reason for the specimen rejection that we found in our study is hemolysis, which accounts for about 22% of the total rejected specimens. In the same manner, a retrospective study conducted at Hera´a General Hospital, Saudi Arabia to find the major cause of pre-analytical errors that caused specimen rejection at the clinical biochemistry department showed that 35% of the total rejection was due to visible hemolysis after centrifugation. In addition, a cross-sectional study conducted in Addis Ababa also noted that the main reason for specimen rejection was hemolysis [12,16]. This could be supported by the fact that inappropriate collection and transfer of specimen to collection tubes and transportation of specimen without centrifugation cause hemolysis.

## Conclusion

The results of our study show that the specimen rejection rate among referred specimens was high, indicating that more interventions are required until the specimen rejection rate reaches the established target. The major reasons for specimen rejection were insufficient specimen volume, hemolysis, and an inappropriate specimen container. Specimen collector training status and experience had a significant association with specimen rejection. Therefore, to minimize specimen rejection rates among referred specimens, all laboratory professionals working at the referring health facilities should be trained in the collection, handling, and transportation of referred tests.

### What is known about this topic

- *Specimen rejection is the result of improper collection, handling, and transportation of specimens;*
- *Specimen rejection causes for delay laboratory analysis and poor patient's health outcome;*
- *High specimen rejection rate is an indicator of poor-quality laboratory diagnosis*.

### What this study adds

- *This study noted the specimen rejection rate and the factors of rejection among referred specimens which has been demonstrated in other settings but had not been demonstrated at Debre Markos Referral Hospital laboratory, Ethiopia*.
